# LPDi GAN: A License Plate De-Identification Method to Preserve Strong Data Utility

**DOI:** 10.3390/s24154922

**Published:** 2024-07-30

**Authors:** Xiying Li, Heng Liu, Qunxiong Lin, Quanzhong Sun, Qianyin Jiang, Shuyan Su

**Affiliations:** 1 School of Intelligent Systems Engineering, Sun Yat-sen University, Shenzhen 518107, China; stslxy@mail.sysu.edu.cn (X.L.); liuh387@mail2.sysu.edu.cn (H.L.); sushy8@mail2.sysu.edu.cn (S.S.); 2 Key Laboratory of Video and Image Intelligent Analysis and Application Technology, Ministry of Public Security, Guangzhou 510006, China; kjksqz@163.com; 3 Guangdong Provincial Key Laboratory of Intelligent Transportation System, Shenzhen 518107, China; 4 School of Computing, Guangzhou Maritime University, Guangzhou 510725, China; jiangqianyin@gzmtu.edu.cn

**Keywords:** de-identification, license plate dataset, data utility, deep learning, generative adversarial networks

## Abstract

License plate (LP) information is an important part of personal privacy, which is protected by law. However, in some publicly available transportation datasets, the LP areas in the images have not been processed. Other datasets have applied simple de-identification operations such as blurring and masking. Such crude operations will lead to a reduction in data utility. In this paper, we propose a method of LP de-identification based on a generative adversarial network (LPDi GAN) to transform an original image to a synthetic one with a generated LP. To maintain the original LP attributes, the background features are extracted from the background to generate LPs that are similar to the originals. The LP template and LP style are also fed into the network to obtain synthetic LPs with controllable characters and higher quality. The results show that LPDi GAN can perceive changes in environmental conditions and LP tilt angles, and control the LP characters through the LP templates. The perceptual similarity metric, Learned Perceptual Image Patch Similarity (LPIPS), reaches 0.25 while ensuring the effect of character recognition on de-identified images, demonstrating that LPDi GAN can achieve outstanding de-identification while preserving strong data utility.

## 1. Introduction

With the advancement of social networks, cloud computing, and data mining technologies, privacy has become a critical social and political issue in the information society, garnering widespread attention [1,2,3,4]. License plate (LP) information, as a crucial component of personal privacy, is legally protected [5]. The European General Data Protection Regulation (GDPR) classifies LP information as personal data that can indirectly identify individuals, with any violation leading to severe penalties [6]. Similarly, China’s Civil Code stipulates that collecting vehicle information without consent constitutes an invasion of privacy [7].

**Figure 1:**
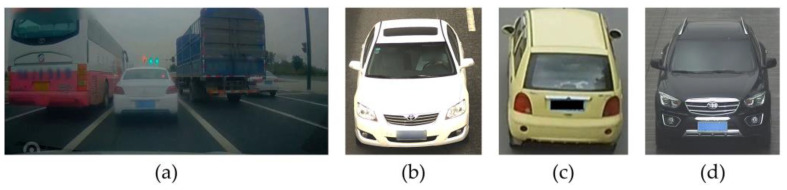
Several public datasets using LP de-identification methods. (**a**) D2-City; (**b**) VRID; (**c**) VeRi; (**d**) CompCars.

Due to legal mandates and the increasing focus on privacy, transportation datasets, such as those for autonomous driving [8,9,10,11,12], vehicle re-identification [13,14,15,16,17], and street-scene understanding [18,19,20,21,22], are considering de-identification to address the LP information involved in the images. Privacy risk levels for images can be categorized into four grades, with the lowest risk being described as images containing no private information [23], also known as complete privacy protection [5]. As depicted in Figure 1, public datasets such as D2-City [24,25], VRID [26,27], VeRi [28], and CompCars [29,30] employ masking and blurring techniques for de-identification, effectively preventing LP information leakage and achieving complete privacy protection. This approach ensures that researchers can use these datasets without risking privacy breaches [31].

However, this simple and effective de-identification method significantly reduces data utility, particularly in tasks such as object detection and character recognition on LPs. Currently, researchers are increasingly focusing on maintaining data utility after de-identification [23,32]. The de-identification method based on adversarial examples aims to preserve data utility by adding visually invisible perturbations [33]. However, it does not fully achieve the goal of de-identification because these adversarial examples can only deceive neural networks and appear identical to the original examples to human observers [34].

With the development of deep learning, generative adversarial networks (GANs) utilize convolutions to learn the characteristics of the original data domain and generate synthetic images with similar distributions [35,36]. Consequently, GANs are widely used in computer vision applications such as data augmentation [37], style transfer [38], and foreground–background fusion [39]. For face generation [40,41], GANs can produce high-quality synthetic faces that do not exist in the real world, which can be utilized for face de-identification [42,43]. However, most face de-identification studies generally overlook background information, and the samples in datasets are usually quite simple [44]. In the transportation field, GANs perform well in advanced technologies such as LP data augmentation [45], super-resolution LP detection [46], and vehicle recoloring [47]. Thus, the question arises: can we use GANs to improve data utility after LP de-identification?

To the best of our knowledge, there are no existing studies on the use of GANs for LP de-identification. This paper aims to address the issue that traditional de-identification methods often overlook data utility. Therefore, an LP de-identification generative adversarial network (LPDi GAN) is proposed. Through the adversarial training process, the generator learns how to convert an LP template to a synthetic LP and progressively enhances the visual quality of synthetic LPs, making them appear realistic and seamlessly integrated with the background. The LPDi GAN also includes an Enhanced Background Perception (EBP) Module and an LP Style Extraction (LPSE) Module, which enable the network to perceive environmental changes, adjust angles, and synthesize data distributions that closely resemble the original. The generated LP, with characters that can be either manually controlled or randomly generated, can be replaced in the original images, ensuring that the LP information cannot be traced back to individuals. Consequently, this method achieves complete privacy protection while significantly preserving data utility after de-identification. The contributions of this paper are as follows:

- **Complete Privacy Protection**: The proposed LP de-identification method (LPDi GAN) achieves complete privacy protection, ensuring that the generated LP information cannot be traced back to individuals.
- **Adaptability and Controllability**: LPDi GAN is adaptable to various LP templates, ambient light conditions, and geometric variations, such as different angles, shapes, and sizes. This adaptability ensures that the method provides controllability of LP characters, background perception, and geometric adjustability.
- **Data Utility Preservation**: Character recognition experiments conducted using the same detector on both the original and de-identified datasets yielded similar results. This indicates that our method effectively preserved the dataset’s utility after de-identification.

## 2. Materials and Methods

### 2.1. Materials

**Figure 2:**
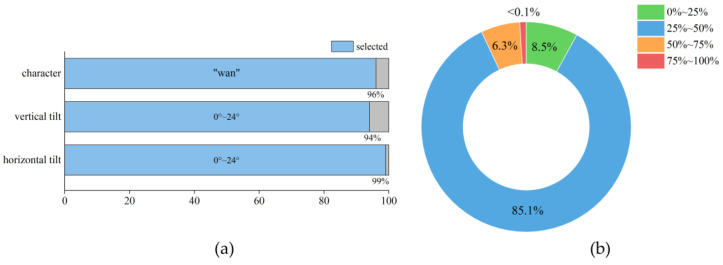
Overview of the CCPD2019 dataset: (**a**) Distribution of characters and vertical and horizontal tilt angles; (**b**) Distribution of LP width relative to sample width.

CCPD2019 is a large traffic dataset that collects roadside parking data for all streets in a provincial capital city in China. The resolution of each image is 720 (width) × 1160 (height) × 3 (channels) and includes extensive labeling information such as characters, locations of the four vertices, and tilt angles of LPs [48]. “ccpd_base” is a sub-dataset of CCPD2019, which has higher quality and is more suitable for our research. In this dataset, 96% of the samples have “wan” as the first character of the LP. This high proportion could cause data imbalance, so we removed samples with other Chinese characters. Similarly, we conducted a statistical analysis of the LP tilt angles, analyzing samples at 5° intervals (as similar angles within the same interval result in similar appearances). For intervals above 24°, each contained fewer than 10,000 samples, and tilt angles exceeding 23.5° were considered outliers because they fell beyond the upper limit of the box plot. To effectively train the generative adversarial network with sufficient samples, we only selected samples with vertical tilt angles from 0 to 24 degrees (94% of the original dataset) and horizontal tilt angles from 0 to 24 degrees (99% of the original dataset). The proportion of selected samples is shown in Figure 2a. Figure 2b also shows the distribution of LP width divided by image width, with ranges of 0%~25%, 25%~50%, 50%~75%, and 75%~100%.

### 2.2. Architecture of LPDi GAN

**Figure 3:**
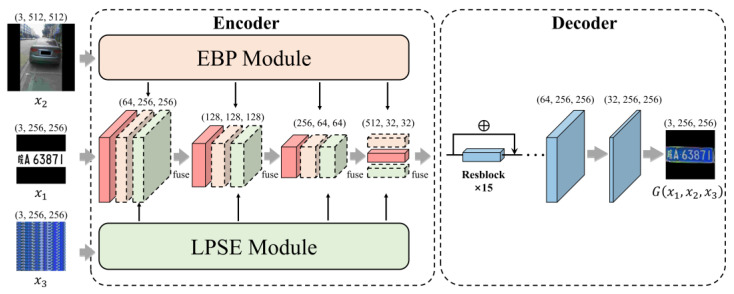
The structure of the generator of LPDi GAN.

**Figure 4:**
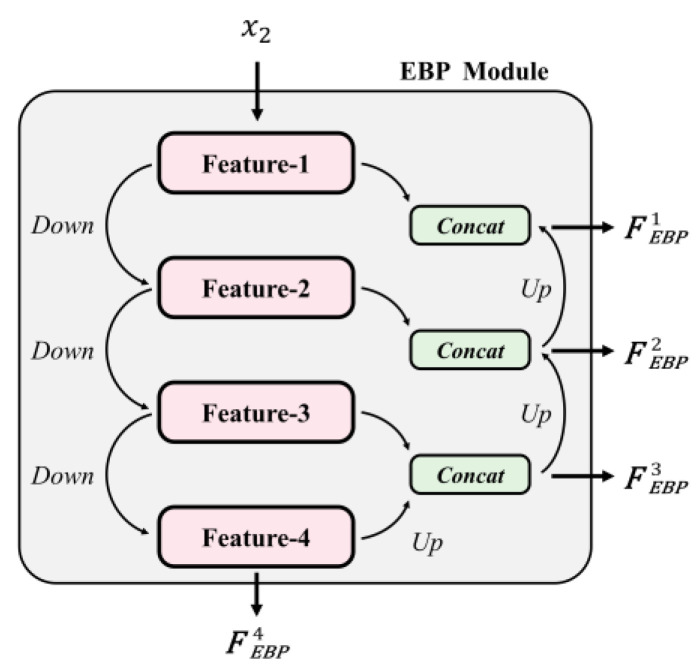
The structure of the EBP Module.

LPDi GAN is proposed to obtain high-quality synthetic LPs for de-identification of transportation datasets. It is composed of a generator and a discriminator, the architectures of which are shown in Figure 3 and Figure 4, respectively.

To help the generator learn to generate LPs more similar to real ones, standard LP templates were constructed to provide information on LP content. Firstly, a standard LP character image set was prepared for each real LP character based on the production standards for LPs. During the training phase, based on the annotated LP information in the dataset, the corresponding standard LP character images were assembled to generate LP templates ($x_{1}$ in Figure 3) according to the standard character distribution layout of real LPs. Despite the use of LP templates, differences in the environment largely affect the texture and color of LPs presented in real images, and the perspective of the shot also leads to the tilt and distortion of LPs. To address this, the non-LP background ($x_{2}$ in Figure 3) was prepared by covering the LP region of the original image with a black mask based on its four vertices’ positions. In addition, a certain number of pixel rows were randomly selected from the original LP to represent the LP style, with the number of rows being one-fifth of the height of the LP region. These pixels were then copied or truncated to resize to a shape of 3×256×256, which is $x_{3}$ in Figure 3. With these three inputs, the encoder can not only learn how to generate LPs that match their content based on the LP templates but also produce LPs with appearance features similar to real ones, using the non-LP background and LP style.

The encoder performs downsampling of the features through convolutions, while the decoder employs transposed convolutions for upsampling. The features of $x_{1}$ are gradually compressed in the encoder, as illustrated by the solid cubes in Figure 3. To consider the background information of the sample and the pixel details of the original LP, the EBP Module and the LPSE Module were integrated into the encoder, with the inputs being non-LP background ($x_{2}$) and LP style ($x_{3}$), respectively. These two modules use convolutional operations for downsampling to extract features from $x_{2}$ and $x_{3}$. However, as the number of convolution layers increases, the receptive field of the output features expands, potentially causing the final output to overly emphasize deeper features. To mitigate this, rather than merging the final output of the two modules with features extracted from $x_{1}$ at the last stage of the encoder, LPDi GAN employs four fusion operations at different stages of downsampling. This strategy allows the encoder to effectively harness both shallow and deep features from $x_{2}$ and $x_{3}$. The four different shapes of features at different stages produced by the EBP Module and LPSE Module are depicted by the dashed cubes in Figure 3. To enable the information interaction of these output features with those extracted from the LP template ($x_{1}$) through downsampling, features at corresponding stages are fused by Equation (1):

(1) $$F^{i+1}=\sigma \left(BN\left(f^{1\times 1}\left(F^{i}+F_{EBP}^{i}+F_{LPSE}^{i}\right)\right)\right),\;i=1,\ldots ,4$$

where i denotes the four stages in the encoder. $F^{i}$, $F_{EBP}^{i}$ and $F_{LPSE}^{i}$ are the features extracted from $x_{1}$, $x_{2}$, and $x_{3}$ at stage i, respectively. At the same stage i, $F^{i}$, $F_{EBP}^{i}$, and $F_{LPSE}^{i}$ have the same shapes. $f^{1\times 1}$ denotes the convolution operation with a 1×1 kernel. The fused features, $F^{i+1}$, are obtained following Batch Normalization (BN) and activation function ReLU (σ). The fused outputs of the first three stages are fed into the subsequent stage, while the final stage’s fused output constitutes the ultimate output, which is then forwarded to the Resblock with skip connections in the decoder. The final synthetic image, $G\left(x_{1},x_{2},x_{3}\right)$, is generated through upsampling.

With input $x_{2}$, the EBP Module mainly focuses on the non-LP background information and the shape of the LP mask. As illustrated in Figure 4, the EBP Module extracts the compressed features (Feature-1 to Feature-4) by downsampling $x_{2}$ using convolutions, which enable the module to capture complex lighting conditions and spatial features. The U-shaped structure effectively captures both high-level and low-level features, facilitating the seamless integration of the synthetic LPs with the background. Each downsampling process can be described as follows:

(2) $$Down\left(x\right)=\sigma \left(BN\left(f_{down}^{3\times 3}\left(x\right)\right)\right)$$

where $f_{down}^{3\times 3}$ denotes the 3×3 convolution kernel with stride 2. However, it is inappropriate for these features (Feature-1 to Feature-3) to be used directly as outputs of the EBP Module, as each layer can only perceive local information, leading to poor global perception. To address this problem, the feature of a deeper layer is upsampled to connect with the feature of the previous layer. For example, $F_{EBP}^{3}$ is obtained by upsampling the deeper Feature-4 and concatenating it with Feature-3. This approach ensures that shallow features have more global information. The equation for upsampling is as follows:

(3) $$Up\left(x\right)=\sigma \left(BN\left(f_{up}^{3\times 3}\left(x\right)\right)\right)$$

where $f_{up}^{3\times 3}$ denotes the 3×3 transposed convolution kernel with stride 2. Finally, the four features ($F_{EBP}^{1}$, $F_{EBP}^{2}$, $F_{EBP}^{3}$, $F_{EBP}^{4}$) are used as the output of the EBP Module.

**Figure 5:**
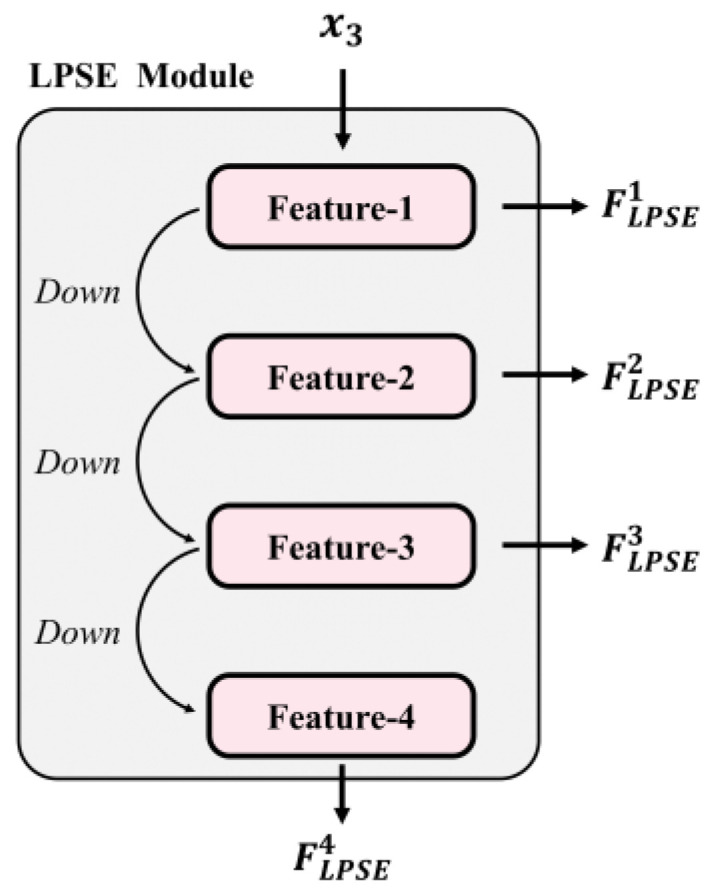
The structure of the LPSE Module.

The LPSE Module primarily realizes the feature extraction of the original LP style ($x_{3}$), as shown in Figure 5. Similar to the EBP Module, input $x_{3}$ is compressed into four features through convolutions, as described in Equation (2). However, since the LPSE Module focuses on the pixel value of the original LP, the amount of information is less compared to the EBP Module. Therefore, the network complexity of the LPSE Module is reduced, resulting in a smaller number of parameters and a faster operation speed. Consequently, these four features are directly used as the output of the LPSE Module.

**Figure 6:**
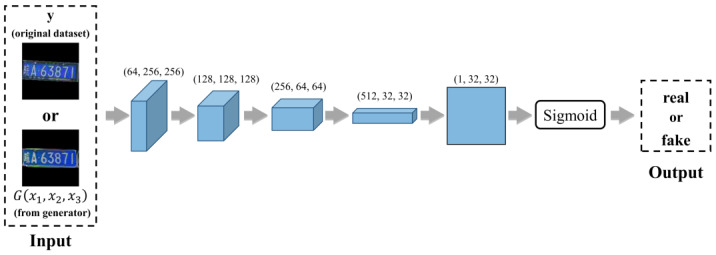
The structure of the discriminator of LPDi GAN.

The primary purpose of the discriminator is to engage in an adversarial game with the generator. The generator creates synthetic images, while the discriminator’s role is to distinguish between real and synthetic images. As illustrated in Figure 6, the discriminator receives two types of inputs: real images from the original dataset (y) and images produced by the generator $G\left(x_{1},x_{2},x_{3}\right)$. These input images are processed through a series of convolutional layers, which progressively extract features at different levels of abstraction. During this process, the spatial resolution of the features decreases while the number of feature channels increases. The extracted features are subsequently mapped to a 32 × 32 patch through further convolutional operations. The final decision of whether an input image is real or synthetic is made based on this patch, and the classification is achieved using the Sigmoid activation function. The discriminator is trained to maximize its accuracy in identifying real images as real and synthetic images as fake, thereby continually challenging the generator to produce more realistic images.

### 2.3. Model Training

In this experiment, the generator G processes the LP template $x_{1}$, non-LP background $x_{2}$, and LP style $x_{3}$ to produce the synthetic image $y^{\prime }$, denoted as $G:\left\{x_{1},x_{2},x_{3}\right\}\to y^{\prime }$. The discriminator D is then used to judge whether the input image is real or fake.

The efficacy of the discriminator in distinguishing between real and synthetic images was evaluated using a binary cross-entropy loss, as depicted in Equation (4). Here, q represents the probability assigned by the discriminator to an input image, ranging from 0 to 1, while p is the ground truth label (0 for fake, 1 for real).

(4) L=−(plogq+(1−p)log(1−q))

It is assumed that the LP template $X_{1}:\left\{x_{1}^{\left(1\right)},\ldots ,x_{1}^{\left(m\right)}\right\}$, the non-LP background $X_{2}:\left\{x_{2}^{\left(1\right)},\ldots ,x_{2}^{\left(m\right)}\right\}$, and the LP style $X_{3}:\left\{x_{3}^{\left(1\right)},\ldots ,x_{3}^{\left(m\right)}\right\}$ are obtained from real samples $X:\left\{x^{\left(1\right)},\ldots ,x^{\left(m\right)}\right\}$. The real LP images $X_{r}:\left\{x_{r}^{\left(1\right)},\ldots ,x_{r}^{\left(m\right)}\right\}$ can be obtained directly from these real samples. During the training of the discriminator, if a sample is derived from the real LP image, $X_{r}$, the ground truth is set to 1. The corresponding loss equation is as follows:

(5) $$-\left(1\cdot \log D\left(x_{r}\right)+\left(1-1\right)\cdot \log\left(1-D\left(x_{r}\right)\right)\right)=-\log D\left(x_{r}\right)$$

Similarly, Equation (6) represents the loss equation in the case that the input is the generator’s synthetic output.

(6) $$-\left(0\cdot \log D\left(G\left(x_{1},x_{2},x_{3}\right)\right)+\left(1-0\right)\cdot \log\left(1-D\left(G\left(x_{1},x_{2},x_{3}\right)\right)\right)\right)=-\log\left(1-D\left(G\left(x_{1},x_{2},x_{3}\right)\right)\right)$$

Therefore, the loss function of LPDi GAN in the process of updating the discriminator is as follows:

(7) $$Loss_{D}=-\frac{1}{m}\overset{m}{\underset{i=1}{\sum }}\left[\log\left(D\left(x_{r}\right)\right)+\log\left(1-D\left(G\left(x_{1},x_{2},x_{3}\right)\right)\right)\right]$$

During the generator’s update phase, the parameters of the discriminator are frozen. The generator’s objective is to deceive the discriminator by producing synthetic images that the discriminator perceives as real, ideally leading to $D\left(G\left(x_{1},x_{2},x_{3}\right)\right)$ approaching the value of 1. The corresponding loss function is given by:

(8) $$Loss_{G}=-\frac{1}{m}\overset{m}{\underset{i=1}{\sum }}\log\left(1-D\left(G\left(x_{1},x_{2},x_{3}\right)\right)\right)$$

Equations (7) and (8) are combined into a composite loss function ${ℒ}_{1}$, as follows:

(9) $${ℒ}_{1}\left(G,D\right)=E_{x_{r}}\left[\log D\left(x_{r}\right)\right]+E_{x_{1},x_{2},x_{3}}\left[\log\left(1-D\left(G\left(x_{1},x_{2},x_{3}\right)\right)\right)\right]$$

To enhance the quality of the synthetic images produced, it is crucial to measure the discrepancy between the generator’s output and the original image. This was achieved by calculating the L1 norm, as shown in Equation (10):

(10) $${ℒ}_{L1}=E_{x_{1},x_{2},x_{3},y}\left[\|y-G\left(x_{1},x_{2},x_{3}\right){\|}_{1}\right]$$

In summary, to describe the adversarial process of the generator and the discriminator, the objective of LPDi GAN can be expressed as follows:

(11) $$G^{*}=\underset{G}{\min}\underset{D}{\max}{ℒ}_{1}\left(G,D\right)+\lambda {ℒ}_{L1}$$

## 3. Results and Discussion

### 3.1. Ablation Study

This paper introduces LPDi GAN, which integrates the EBP Module and LPSE Module. To evaluate the contribution of these modules, a baseline model devoid of the EBP Module and the LPSE Module was established. An ablation study was conducted to further demonstrate the effectiveness of different modules, with the results presented in Table 1. The models involved in the experiments included the baseline model, the model with only the EBP Module (baseline + EBP), the model with only the LPSE Module (baseline + LPSE), and the complete LPDi GAN. The LPIPS metric based on deep features was used to evaluate model performance, as it can accurately reflect the perceptual similarity between two images. The experiment measured the perceptual similarity between the de-identified datasets created by the various models and the original datasets. A lower LPIPS value indicated a higher perceptual similarity between the two images. The experimental results showed that the baseline model performed the worst. Introducing the EBP Module or the LPSE Module significantly improved the perceptual similarity between the datasets. The simultaneous use of both modules yielded the best results, with the lowest LPIPS value of 0.25, demonstrating that the de-identified dataset generated by LPDi GAN more closely mirrored the distribution of the original dataset. These results validated the effectiveness of the EBP Module and the LPSE Module.

**Figure 7:**
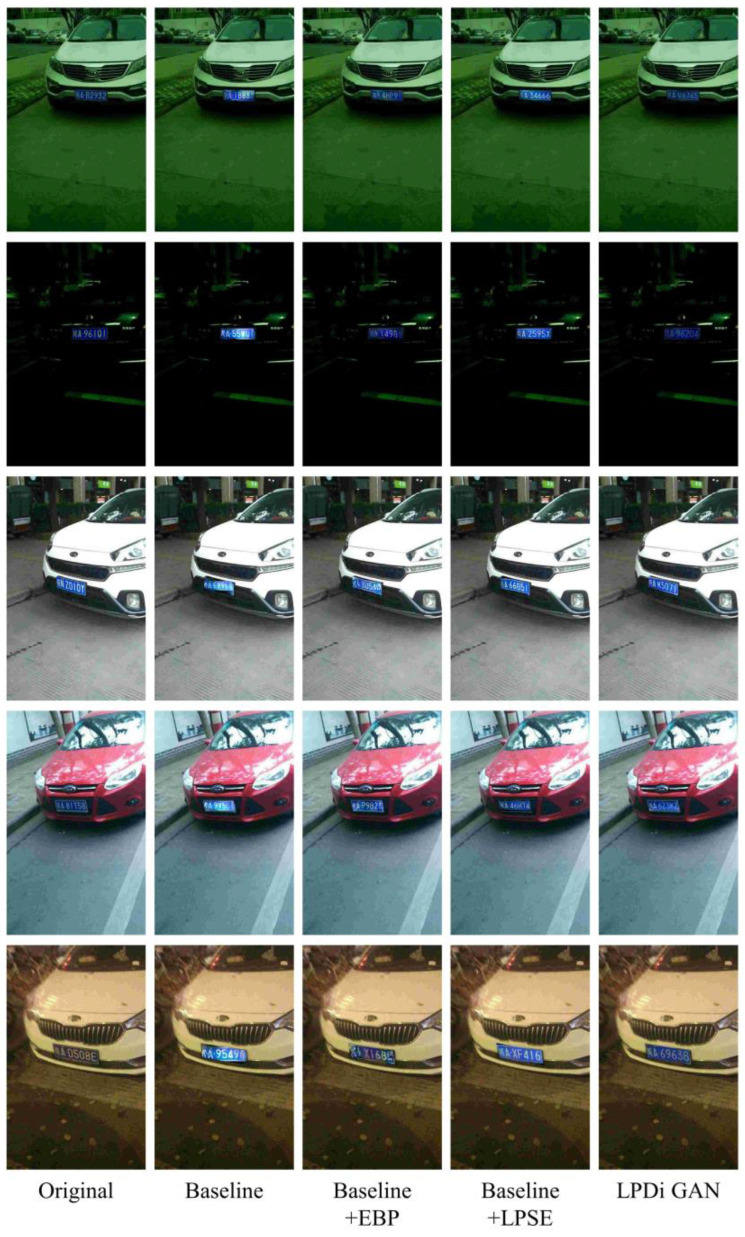
Visualization of the generated images using different modules. The first column shows the original image. The second column shows images generated through the baseline model, which does not include the EBP Module and the LPSE Module. The third column shows images generated through the baseline model with the EBP Module. The fourth column shows images generated through the baseline model with the LPSE Module. The fifth column shows images generated through LPDi GAN.

Additionally, Figure 7 presents several samples to further illustrate the effects of the two modules on the visual outcomes of synthetic LPs. In Figure 7, every column of images is generated by different models. In the second column of Figure 7, the images generated by the baseline model (without the EBP Module and the LPSE Module) exhibit a distinct horizontal alignment tendency, lacking the necessary perspective distortion that is crucial for seamless integration with the background. Furthermore, the characters in these images suffer from abnormal distortion and blurriness, which significantly reduces the quality of the synthetic LPs. By incorporating the EBP Module into the baseline model (as shown in the third column of Figure 7), the synthetic LPs achieved significant advancements in angle adjustment, basically matching the tilt angles of the original LPs. This indicated that the EBP Module enabled the generator to learn the angular information of the LPs. Additionally, the EBP Module effectively reduced the brightness discrepancies between the synthetic LPs and the background. However, there still existed the issue of distorted and blurry character content. Integrating the LPSE Module into the baseline model (as depicted in the fourth column of Figure 7) resulted in a noticeable improvement in the quality of character generation in the synthetic images, presenting clearer and more accurate character content. Yet the issues of abnormal lighting and perspective distortion persisted, particularly in the first, second, and fifth row of the fourth column. LPDi GAN integrated both modules, which not only excelled in the quality of character generation but also demonstrated superior capability in angle adjustment and background perception.

### 3.2. Effectiveness of Network Structure

The elaborate LPDi GAN is capable of generating synthetic images with specified characters based on the content of the LP template. The EBP module enhances the integration of the synthetic LPs with the background by perceiving environmental lighting and the original LPs’ geometric features, while the LPSE module improves the quality of the characters on the synthetic LPs by learning the features of the original LPs. The experiments below evaluated the controllability of character generation, background perception, angle adjustment capability, and adaptability to different LP sizes, demonstrating the effectiveness of the LPDi GAN structure.

#### 3.2.1. Controllability of Character Generation

**Figure 8:**
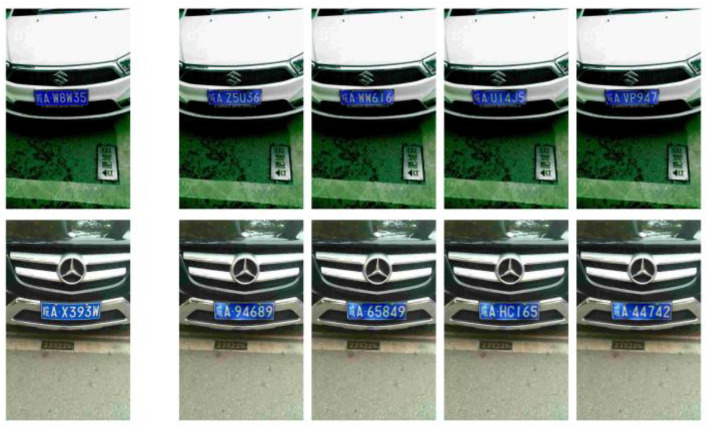

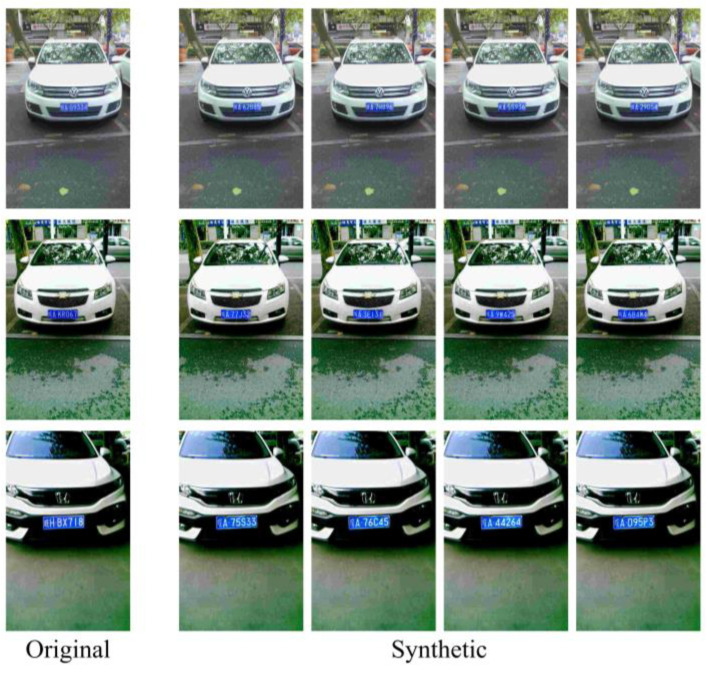
The results of controlling LP: different LP characters are synthesized in the same background. The first column shows the original images. The rest are the synthetic images generated with different LP templates.

When de-identifying the LP in a sample using LPDi GAN, an LP template with a random combination of characters is used as input to control the characters of the synthetic LP. Figure 8 shows some examples of the generated images. The results indicated that LPDi GAN successfully generated the corresponding LP characters, producing clear synthetic images for each random LP template input. This proved that the character generation was controllable and that the LP template mechanism in LPDi GAN was rational.

Additionally, an experiment was conducted to further demonstrate the controllability of character generation by LPDi GAN. First, 10,000 test samples were randomly selected to apply LPDi GAN to generate synthetic LPs based on random LP templates. Then, the characters of the synthesized plates were compared with the corresponding templates. The results showed that among the 10,000 synthetic LPs, only 141 had character discrepancies with their corresponding templates. In these 141 samples, 135 synthetic LPs had a single character error, and the remaining 6 LPs had two character errors. Therefore, LPDi GAN successfully generated characters for 98.6% of the test samples, proving the controllability of character generation in LPs by LPDi GAN.

#### 3.2.2. Background Perception

**Figure 9:**
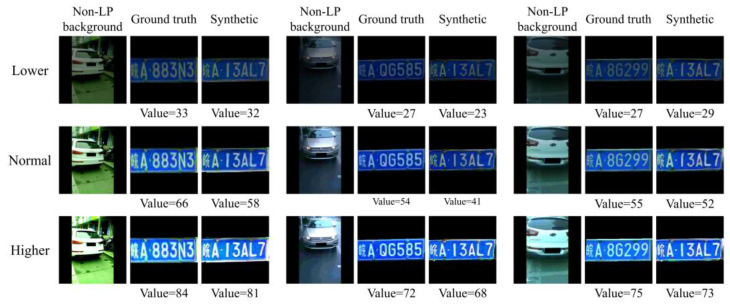
Comparison of the value of ground truth and synthetic LPs under different lighting conditions.

Background perception is mainly reflected in the sensitivity to the illumination of the composite image, as the consistency of the overall environment makes the image more natural and realistic. In the HSV color space, the V channel (value) indicates the brightness of the image. In this part of the experiment, the value of the sample was adjusted to two other levels, lower and higher, by multiplying the original value by a conversion factor. The value of the original sample represented the normal level, while the lower and higher levels referred to conversion factors of 0.5 and 1.5, respectively. The values of the original LP and the generated LP were calculated, and the experimental results of several samples are shown in Figure 9. Each row shows a set of images under one kind of environmental lighting level. Among them, the values of the synthetic image and the original image are very close to each other, and their illumination appears similar when viewed by the human eye.

In order to better demonstrate the ability to perceive the background information of the whole dataset, the values of all samples were calculated. Table 2 shows the mean value of synthetic and original images at three levels. The mean value of the de-identification dataset and the original dataset were almost the same at the lower level and normal level, differing by 3 and 1, respectively. The difference in the mean value at the higher level was less than 10, which was considered equally excellent, although slightly larger than others. These results indicated that LPDi GAN has the ability to perceive and adapt to the background lighting conditions.

#### 3.2.3. Angle Adjustment Capability

**Figure 10:**
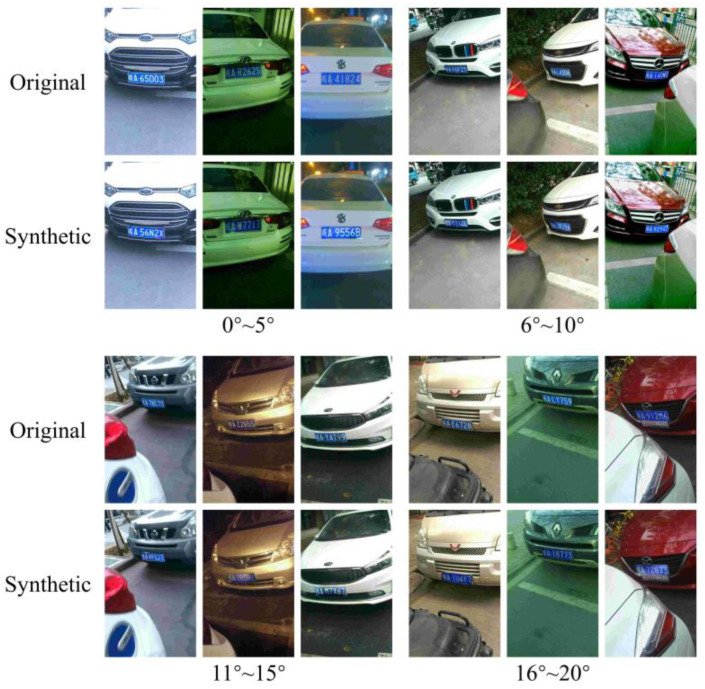
Comparison of original and synthetic LPs at different ranges of tilt angles.

The input of the EBP Module, the non-LP background, includes the mask information of the LP area, providing the geometric features of the LP. This enables the generator to learn and synthesize the corresponding tilt and perspective angles of the LP. The synthetic images and the corresponding original images at different ranges of tilt angles are shown in Figure 10. The synthetic images successfully adjusted the tilt angle of the LP while maintaining the clarity of the characters, demonstrating that LPDi GAN has the capability to adjust the angle of LPs.

#### 3.2.4. Adaptability to Different Size

**Figure 11:**
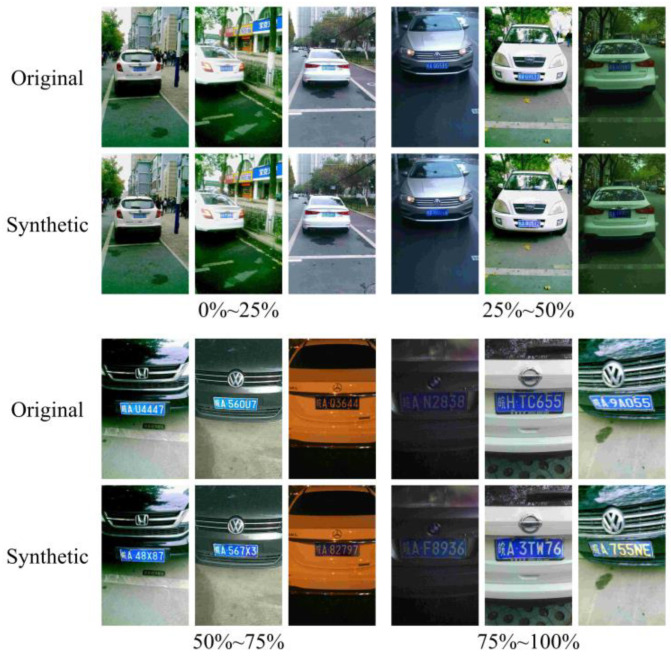
Comparison of original and synthetic images with different width ratios.

The width ratio of the LP to the image reflects the size of the LP in the image. Based on this ratio, the dataset could be divided into four size ranges: 0%~25%, 25%~50%, 50%~75%, and 75%~100%. Figure 11 shows the results of LPDi GAN for different LP sizes. In each section, the first row contains the synthetic images, and the second row contains the original images. The results indicated that LPDi GAN can effectively handle diverse LP sizes and successfully create synthetic LPs that maintain fidelity to their original dimensions.

### 3.3. Evaluation of Similarity

**Figure 12:**
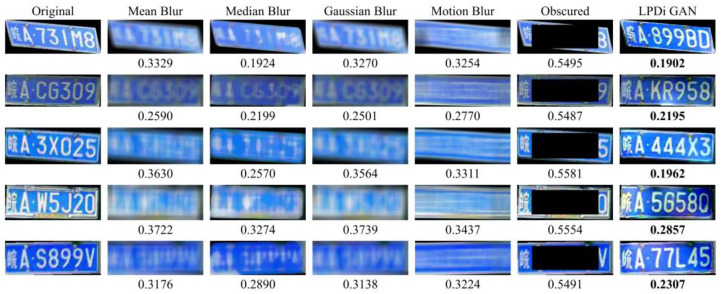
Comparison of LPIPS values for different LP de-identification methods.

To substantiate the high perceptual similarity between the original dataset and the de-identified images generated by LPDi GAN, a comparative analysis was conducted using the LPIPS metric against several other existing LP de-identification methods. Figure 12 presents some examples from various de-identification techniques, with the LPIPS metric calculated individually for each sample. Notably, the LPIPS values of LPDi GAN were the lowest among the compared methods. This indicated that LPDi GAN achieved the most effective perceptual similarity between the synthesized and original images. Furthermore, the actual effects of some de-identification methods were not ideal because the content of the original LPs could still be roughly discerned by the human eye, for example, the samples treated with the Gaussian Blur method in the first row and the Mean Blur method in the second row. Thus, these LP de-identification methods failed to provide complete privacy protection.

For a comprehensive and precise assessment of the perceptual similarity across these methods, the mean LPIPS values for the entire dataset were calculated and are presented in Table 3. The results clearly demonstrated that the proposed LPDi GAN holds an absolute advantage with the lowest LPIPS value of 0.25. LPDi GAN not only ensured complete privacy protection but also maintained an exceptional level of perceptual similarity between the synthetic images and the original images.

### 3.4. Data Availability

Character recognition experiments were designed to verify whether the utility of the original dataset was preserved in the de-identified dataset generated from LPDi GAN. An efficient character recognition network, CRNN, was trained on the original and de-identified datasets independently. The results of the character recognition experiments are detailed in Table 4. The accuracy rates for the test sets derived from the original and de-identified datasets were 97.16% and 97.62%, respectively, which were very close. The results indicated that the de-identified dataset maintained a high degree of utility similar to that of the original dataset, ensuring that the de-identification process did not significantly compromise the dataset’s practical applicability.

### 3.5. Practicality of the Proposed Method

**Figure 13:**
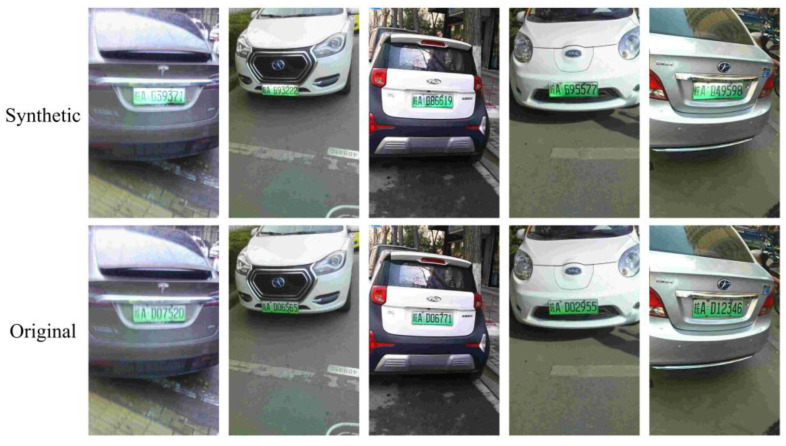
De-identification results of LPDi GAN on CCPD2020 dataset.

In the CCPD2019 dataset, LPs featuring white characters on a blue background are typically associated with traditional fuel vehicles. With the advent of new energy vehicles, the scope of LPDi GAN can be extended to the CCPD2020 dataset. This dataset includes LPs with black characters set against a dynamic white and green gradient background. Figure 13 presents the de-identification results on CCPD2020, with the first row showing the synthetic images and the second row showing the corresponding original images. The characters in the synthetic LP images are clear, and the LP background’s green and white gradient is similar to the original LP. This demonstrated that LPDi GAN is not only effective for LPs of traditional fuel vehicles but also adaptable to the unique characteristics of LPs of new energy vehicles.

## 4. Conclusions

In this paper, a novel LP de-identification method, LPDi GAN, is proposed to protect LP privacy in datasets. The LP characters of the synthetic images can be controlled using LP templates, allowing for manual editing of the characters. The network also enhances background perception using the EBP Module and improves the quality of generated characters using the LPSE Module, making synthetic images perceptually similar to the real images. The experimental results demonstrate that our method, which ensures complete privacy protection, produces de-identified datasets with high data utility. Consequently, our method can be applied to a wide range of transportation datasets, allowing scholars to conduct various research tasks without concern about the impact of de-identified data on task outcomes.

In this study, we explored the angle adjustment capability of LPDi GAN. However, due to the limitations of the dataset, it was challenging to verify whether the proposed method can work effectively under large tilt angles. In future work, we plan to create a large-scale dataset with large tilt angles and improve the network to learn these features, enabling the de-identification process to adapt to more diverse application scenarios.

Additionally, we recognize the potential misuse of our method for malicious purposes. Therefore, deepfake detection of LPs is a planned follow-up task. Through LPDi GAN, we can generate de-identified datasets based on the original datasets, providing valuable data resources for LP deepfake detection. Utilizing these data, we can explore the domain similarities and differences between the two datasets and develop a deep learning model specifically for LP deepfake detection. This will provide robust support for combating and preventing illegal activities involving deepfake technology, thereby enhancing security measures and preventing the misuse of deepfake technology in criminal activities.

## Figures and Tables

**Table 1:** The results of LPIPS using different combination of the EBP Module and the LPSE Module.

	Baseline	Baseline + EBP	Baseline + LPSE	LPDi GAN
LPIPS	0.33	0.26	0.26	**0.25**

**Table 2:** Average value of all samples in the original and de-identification datasets under different lighting conditions.

Level	Average Value	
	Synthetic	Ground Truth
Lower	70	73
Normal	58	59
Higher	37	29

**Table 3:** Average LPIPS values for different LP de-identification methods.

	Mean Blur	Median Blur	Gaussian Blur	Motion Blur	Obscured	Proposed
LPIPS	0.34	0.27	0.34	0.34	0.55	**0.25**

**Table 4:** LP character recognition results for the original and the de-identification dataset.

	Original Dataset	De-Identification Dataset
Accuracy	97.16%	97.62%

## Data Availability

Data are contained within the article.
